# Hymeneal

**Published:** 1885-12-20

**Authors:** 


HYMENIAL.—Married, on Thursday, December 13, 1885, at the First Presbyterian Church, in Atlanta, by Rev. Dr. Barnett, Miss KATE GASTON and Dr. EDWARD N. SHAW. The bride is the accomplished daughter of Professor J. McF. Gaston, the eminent Surgeon, of the Southern Medical College, of Atlanta, and the groom is a worthy and intelligent young physician of Texas.

